# Body image dissatisfaction and disordered eating behaviors among female students at Birzeit university: a cross-sectional study

**DOI:** 10.1186/s40337-026-01698-4

**Published:** 2026-07-08

**Authors:** Shahed Mohammad, Shahad Hassan, Mawada Zeyad, Yezan Mohammad, Mohanad Samaheen, Yousef Alnajjar, Zahra Makhamreh, Maen Mohammad

**Affiliations:** 1 Nutrition and Dietetics Department, Faculty of Pharmacy, Nursing and Health Professions, Birzeit University, Birzeit, Palestine; 2 Faculty of Medicine, Al-Quds University, Jerusalem, Palestine; 3 Faculty of Medicine, An-Najah Univeristy, Nablus, Palestine; 4 Internal Medicine Department, Al-Makassed Islamic Charitable Hospital, Jerusalem, Palestine; 5 Faculty of Medicine, Palestine Polytechnic University, Hebron, Palestine

**Keywords:** Eating disorders, Body image dissatisfaction, Mental health, BMI.

## Abstract

**Background:**

Body image dissatisfaction (BID) is a growing psychosocial concern and is strongly associated with disordered eating behaviors (DEBs), especially among young women. Although global awareness of these issues is increasing, research exploring the prevalence and relationship between BID and DEBs in Palestine remains scarce. This study aimed to assess the prevalence of DEBs and examine their association with BID among female university students.

**Methods:**

A cross-sectional study was conducted at Birzeit University from February to June 2025 among 295 female students aged 18–25 years recruited through convenience sampling. Data were collected using a structured, self-administered questionnaire that included sociodemographic and anthropometric characteristics, the Eating Attitudes Test-26 (EAT-26), and the Body Shape Questionnaire-16B (BSQ-16B). DEB risk was defined as an EAT-26 score ≥ 20.

**Results:**

A total of 12.9% of participants were identified as being at risk of DEBs. BSQ-16B and EAT-26 scores were moderately positively correlated (*r* = 0.535, *p* < 0.001). DEBs were significantly associated with higher BMI, smoking, previous DEBs, social media influence, binge eating, weight-loss attempts, and a positive BMI discrepancy between actual and ideal BMI.

**Conclusion:**

This study found that a notable proportion of Palestinian university women were at risk of DEBs and that BID was moderately associated with DEBs. These findings support the importance of screening for disordered eating behaviors and body image concerns among female university students in Palestine.

## Abstract (plain language summary)

This study looked at how young women at Birzeit University feel about their bodies and how these feelings may be linked to unhealthy eating habits. We surveyed 295 female students aged 18 to 25 and asked them about their eating patterns, weight-control behaviors, and how they see their body shape. About 13% of the students showed signs that they may be at risk of disordered eating. Students who felt unhappy with their body shape were more likely to report unhealthy behaviors such as strict dieting, skipping meals, binge eating, or using unsafe methods to lose weight. Those who believed their “ideal” weight was lower than their current weight were also more likely to be at risk. Social media and higher body weight were linked to greater body dissatisfaction. Although not all students were at high risk, many still reported unhealthy weight-control practices. These findings highlight the importance of promoting positive body image, healthy lifestyle habits, and early support services for young women at universities in Palestine.

## Background

Body image dissatisfaction (BID) is increasingly seen as a key factor in the development of eating disorders, which greatly affect individuals’ physical health, psychological well-being, and quality of life [1]. Body image is a complex concept that includes one’s perceptions, thoughts, feelings, and behaviors toward the body [1]. BID occurs when there is a perceived gap between one’s actual and ideal body shape [1]. This dissatisfaction can lead to harmful behaviors like restrictive dieting, fasting, excessive exercise, and purging [2]. When these behaviors persist, they may develop into clinical eating disorders, which are among the most dangerous psychiatric conditions because of their complicated medical and psychological effects [2–4].

Globally, eating disorders and BID are widespread public health issues, especially among adolescents and young adults [1]. In Western countries, the spread of thin-ideal body standards, boosted by media and digital platforms, has contributed to increased body dissatisfaction and eating disorder risk, particularly among young women. Yet, many individuals affected remain undiagnosed due to limited screening, stigma, and inadequate clinical training. These obstacles prevent timely intervention and treatment [5, 6]. Consequently, many affected people do not get prompt help.

While once considered culture-bound to the West, eating disorders are now increasingly documented in non-Western regions. Cross-cultural studies suggest that globalization, urbanization, and the spread of Western media ideals are reshaping traditional beauty standards [7]. In many Arab countries, including Palestine, a historical preference for curvaceous figures is being replaced by ideals favoring thinness [7, 8]. On the other hand, some findings contrast with this trend. For example, a study conducted in Iran found that women interested in Western culture were more likely to be satisfied with their body shape. Despite geographic proximity, countries within the Middle East differ substantially in cultural norms, religious practices, family structures, media environments, and sociopolitical realities, all of which may shape body image perceptions and eating-related behaviors differently [9]. These variations indicate that a complex interplay of sociocultural and psychological factors influences eating disorders.

Cultural and social dynamics, including family norms, peer influences, and sociopolitical pressures, interact with individual factors like body mass index (BMI) and self-esteem to shape vulnerability to eating disorders [10]. Social media, in particular, plays a powerful role by curating and reinforcing unrealistic body ideals. Research has consistently shown that greater social media use is linked with higher levels of BID and disordered eating, especially among young women [11]. Furthermore, individuals with higher BMI often experience intensified pressure to conform to societal body ideals, exacerbating the risk of unhealthy weight-control practices [12].

Female university students are especially vulnerable. Academic stress, social comparisons, and the desire to conform to peer and media-driven standards increase the risk. International studies consistently report higher rates of BID and DEBs among college women [13]. In the United Arab Emirates, for example, nearly one-quarter of female students exceeded the clinical threshold on the EAT-26, and three-quarters were dissatisfied with their body image [14]. Disordered eating attitudes in that group were strongly linked to body dissatisfaction [14]. Similar patterns are observed across Europe, Asia, and North America, highlighting the global scope of the problem [5].

Despite increasing awareness, research on disordered eating behaviors and body image in Palestine remains limited. Most available evidence in this field comes from Western populations, while data from Palestinian university students are scarce. To our knowledge, no previous study has specifically examined the association between body image dissatisfaction and disordered eating behaviors among Palestinian female university students. Therefore, this study aimed primarily to estimate the prevalence of DEBs and secondarily to examine their association with BID and BMI discrepancy among female students at Birzeit University. By identifying these patterns, the study aims to highlight an underexplored public health concern in this population.

## Methods

### Study design, population, and sampling

A cross-sectional study was conducted between February and June 2025 among undergraduate female students at Birzeit University. Participants were recruited through convenience sampling across various academic departments. The primary outcome was the prevalence of DEBs as identified by EAT-26, while secondary analyses examined associations between DEBs, BID, BMI category, and BMI discrepancy.

The required sample size was calculated using Cochran’s formula, assuming a disordered eating prevalence of 10–20% [15, 16], a 95% confidence level (Z = 1.96), and a 5% margin of error (d = 0.05). The estimated minimum sample size was 246 participants. A total of 295 students were included in the final analysis. The sample size calculation was intended to estimate DEB prevalence and was not specifically powered for inferential analyses examining associations between BID and DEBs. The survey link was distributed electronically through university channels using convenience sampling. Because the link was shared broadly and anonymously, the exact number of students who received the invitation could not be determined; therefore, a formal response rate could not be calculated. This is acknowledged as a limitation.

Inclusion criteria were: female students aged 18–25 years, currently enrolled in any academic program, capable of providing informed consent, and willing to complete a self-administered questionnaire. The age range of 18–25 years was selected to capture the typical undergraduate age group and a developmental stage commonly examined in research on body image and disordered eating behaviors. Eligibility was screened at the beginning of the Google Form through direct yes/no questions addressing age, enrollment status, pregnancy, breastfeeding, presence of children, chronic illnesses affecting weight or eating, medically prescribed diets, and physical disabilities that might affect body image perception. Participants who met any exclusion criterion were not included in the final analysis. Exclusion criteria included pregnancy, breastfeeding, having children, chronic illnesses affecting weight or eating (e.g., diabetes, hypothyroidism, polycystic ovary syndrome), medically prescribed diets (e.g., for celiac disease or peptic ulcers), or physical disabilities that might alter body image perception.

### Data collection measurements and tools

Data were collected using a structured, self-administered questionnaire composed of two validated instruments. Initially piloted in paper format to assess clarity and comprehension, the final version was distributed electronically via a Google Form in Arabic.

The questionnaire comprised three main sections: The first section captured sociodemographic, anthropometric, lifestyle, and environmental factors. These included age, academic year, marital status, residency, self-reported height, weight, and perceived ideal weight. Lifestyle variables encompassed physical activity, sleep duration, smoking status, employment status, medical history, surgical history, and current medication use. Environmental factors such as family and peer pressure, as well as the perceived influence of social media on body image, were also assessed. Participants rated the perceived impact of social media on their body shape and appearance using a single study-specific item on a 6-point Likert scale (1 = never to 6 = always). Perceived stress was assessed using a single self-rated item with three response options (low, moderate, high). These items were included for exploratory purposes and were not derived from a validated scale.

The second section addressed weight-loss behaviors using two direct questions added by the research team: (1) “Have you attempted to lose weight during the past year?” (yes/no), and (2) “If yes, which methods did you use?” with multiple-response options including dieting, physical activity, herbs, medications, and skipping meals. It also assessed disordered eating behaviors using a validated instrument called the Eating Attitudes Test-26 (EAT-26) [17]. The EAT-26 consists of 26 items scored on a 6-point Likert scale (Always = 3, Usually = 2, Often = 1; Sometimes, Rarely, and Never = 0), with the final item reverse-scored. A total score ≥ 20 indicates risk for disordered eating. In line with EAT-26 guidelines, five additional behavioral items were included to assess extreme weight control practices (e.g., binge eating, self-induced vomiting, laxative use, excessive exercise, and unintentional weight loss > 9 kg in the past six months). Though not part of the total EAT-26 score, any positive response to these items suggests a possible risk of an eating disorder and the need for further clinical evaluation. The Arabic version of EAT-26 used in this study was previously validated in the Arab Journal of Psychiatry (1994), supporting its reliability and cultural relevance [18].

The third section assessed body image dissatisfaction which was evaluated using the shortened Body Shape Questionnaire (BSQ-16B) [19]. The BSQ-16B consists of 16 items scored on a 6-point Likert scale ranging from 1 (never) to 6 (always), yielding a total score range from 16 to 96, with higher scores indicating greater body-shape dissatisfaction over the previous four weeks. The BSQ-16B was translated into Arabic and culturally adapted by the research team. The translation process involved initial forward translation, review of wording for linguistic and cultural appropriateness, and pilot testing to ensure clarity and comprehension among the target population. Minor wording modifications were made to improve semantic clarity while preserving the original scoring structure and conceptual meaning.

### Ethical considerations

Ethical approval for this study was granted by the Birzeit University Ethical Committee. The purpose of the study was clearly explained to all participants, and participation was completely voluntary. Informed consent was obtained before data collection, and participants were assured they could withdraw at any time without penalty. To protect confidentiality, no identifying information was collected. All responses were anonymized, securely stored, and only accessible to the research team.

### Statistical analysis

Data were analyzed using IBM SPSS Statistics version 23 and R (Rstudio). Normality was assessed with histograms. Continuous variables were presented as mean ± standard deviation or median (interquartile range), and categorical variables as frequencies and percentages. Disordered eating risk was defined as an EAT-26 score of 20 or higher. Group differences were evaluated using chi-square tests for categorical variables and independent-sample t-tests for continuous variables, while the Pearson correlation measured the relationship between BSQ and EAT-26 scores. Body mass index (BMI) was calculated from self-reported height and weight. BMI categories were defined using standard WHO cutoffs: underweight (< 18.5 kg/m²), normal weight (18.5–24.9 kg/m²), overweight (25.0–29.9 kg/m²), and obesity (≥ 30.0 kg/m²). BMI discrepancy (actual minus perceived ideal BMI) was calculated, with positive values indicating a desire for weight loss and negative values indicating a desire for weight gain.; its association with EAT-26 and continuous BSQ scores was explored through cross-tabulations and chi-square tests. Statistical significance was set at *p* < 0.05.

## Results

A total of 295 female students were included in the analysis. Their mean age was 20.4 ± 1.6 years, and the mean BMI was 23.0 ± 4.1 kg/m². Most participants were single, lived with their families, and were classified as normal weight. The prevalence of DEBs was 12.9%, (Tables 1 and 2)

**Table 1:** Sociodemographic, anthropometric, health, and psychological scale scores (*N* = 295)

Characteristic	Value
Sociodemographic characteristics	
Age, years (mean ± SD)	20.4 ± 1.6
Anthropometrics	
BMI, kg/m² (mean ± SD)	23.0 ± 4.1
Health-related	
Smoking status (yes)	12.2%
History of disordered eating (yes)	18.0%
Psychological scales (continuous)	
BSQ score (median [IQR])	36.0 [25.0–53.0]
EAT-26 score (median [IQR])	8.0 [6.0–15.0]

Values are percentages unless otherwise indicated. Continuous variables are shown as mean ± SD or median [IQR]

**Table 2:** Summary of disordered-eating behaviors, sleep duration, and social media influence

Characteristic	Value
Disordered eating behaviors (past 6 months)	
Binge eating (≥ 2–3×/month)	25.4%
Vomiting	17.6%
Laxatives/diet pills	18.3%
Excessive exercise (> 60 min/day)	2.4%
Sleep duration	
4–5 h	24.1%
6–7 h	59.7%
≥8 h	14.9%
Social media influence	
Never	13.2%
Rarely	14.2%
Sometimes	40.7%
Often	21.0%
Always	10.8%

Values are percentages of total participants (*N* = 295)

Table 2 summarizes lifestyle, disordered eating behaviors, and social influences. Nearly one-quarter of participants reported binge eating episodes (≥ 2–3 times per month) in the past six months, while 17.6% had engaged in vomiting and 18.3% in laxative or diet pill use. Excessive exercise (> 60 min/day) for weight control was less common (2.4%). Most students reported sleeping 6–7 h nightly (59.7%). Regarding social media, 40.7% stated it sometimes influenced their body image, and 31.8% reported frequent or constant influence.

Approximately two-thirds of students were classified as normal weight, with smaller proportions in the underweight, overweight, and obese categories. Dieting and physical activity were the most common weight-control methods in the past year, whereas meal skipping, herbal products, and medications were less frequently reported. Overall, most participants reported walking more than engaging in vigorous physical activity.

**Fig. 1:**
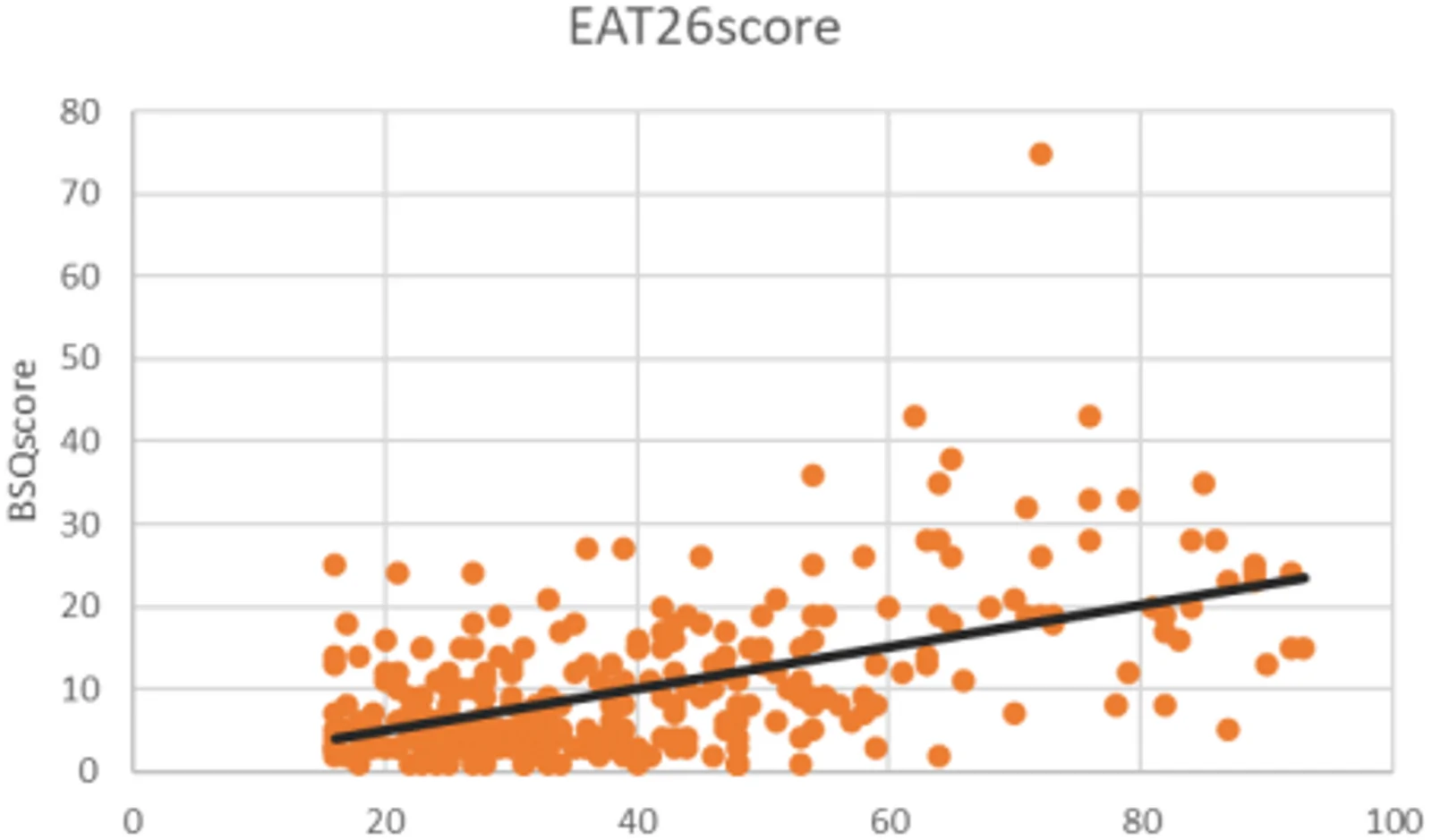
Scatter plot showing the linear positive relationship between EAT26 and BSQ16 scores

A significant moderate positive correlation was observed between BSQ-16B and EAT-26 scores (*r* = 0.535, *p* < 0.001), indicating that higher body-shape dissatisfaction was associated with greater DEB risk (Fig. 1). Overall, 12.9% of students (*n* = 38) were categorized as being at risk of disordered eating based on EAT-26 ≥ 20. Table 3 displays bivariate associations. Compared with their peers, at-risk students were significantly more likely to fall into overweight and obese BMI categories (*p* < 0.001), to report a history of disordered eating (*p* = 0.019), and to be current smokers (*p* = 0.021). Under the section of weight-related behaviors, risk-group students were substantially more likely to report weight-loss attempts in the past year (*p* = 0.001), including dieting, use of herbs, medications, and meal skipping (all *p* ≤ 0.005). They were also more likely to binge eat (39.5% vs. 23.3%, *p* = 0.033) and to engage in prolonged daily exercise for weight control (7.9% vs. 1.6%, *p* = 0.017). In addition, BMI discrepancy (actual > ideal) was associated with disordered eating risk (*p* = 0.028).

**Table 3:** Bivariate associations with disordered eating risk (EAT-26 ≥ 20), including BMI discrepancy

Study variable	Positive risk of DE (*N* = 38)	Negative risk of DE (*N* = 257)	*P*-value
Age (years), mean ± SD	20.55 ± 1.655	20.43 ± 1.550	0.915 a
BMI categories			
Underweight	4 (10.5%)	25 (9.7%)	
Normal weight	14 (35.8%)	176 (68.5%)	
Overweight	14 (36.8%)	47 (18.3%)	
Obese	6 (15.8%)	9 (3.5%)	
Study year			
Second year	6 (15.8%)	50 (19.5%)	0.966 C
Third year	15 (39.5%)	91 (35.4%)	
Fourth year	7 (18.4%)	54 (21.0%)	
Fifth year	5 (13.2%)	25 (9.7%)	
Residence type			
University dorm	5 (13.2%)	20 (7.8%)	0.535 C
With family	31 (81.6%)	224 (87.2%)	
Residence			
City	23 (60.5%)	99 (38.6%)	0.535 C
Village	10 (26.3%)	121 (47.1%)	
Marital status			
Single	33 (86.8%)	231 (89.8%)	0.693 C
Engaged	4 (10.5%)	15 (5.8%)	
Married	1 (2.6%)	11 (4.2%)	
Eating disorder history (yes)	9 (23.7%)	44 (17.1%)	
Smoking status (yes)	9 (23.7%)	27 (10.5%)	
Employment: don’t work	37 (97.4%)	226 (87.9%)	0.178 C
Sleep hours			
4–5 h	14 (36.8%)	57 (22.2%)	0.322 C
6–7 h	18 (47.4%)	158 (61.5%)	
≥8 h	6 (15.8%)	38 (14.8%)	
Stress level			
Moderate	14 (36.8%)	126 (49.0%)	0.171 C
High	24 (63.2%)	124 (48.2%)	
Social media effect			
Sometimes	15 (38.5%)	105 (40.9%)	0.017 *C
Often	13 (34.7%)	49 (19.1%)	
Always	7 (18.4%)	25 (9.7%)	
Weight-related behaviors			
Weight loss attempts during past year (yes)	32 (84.2%)	130 (50.8%)	0.001 *C
Weight loss diet (yes)	28 (73.7%)	98 (38.1%)	0.000 *C
Weight loss physical activity	21 (55.3%)	82 (31.9%)	0.005 *C
Weight loss herbs (yes)	11 (28.9%)	16 (6.2%)	0.000 *C
Weight loss medication (yes)	6 (15.8%)	5 (1.9%)	0.000 *C
Weight loss skipping meals (yes)	13 (34.2%)	19 (7.4%)	0.000 *C
Binge eating ≥ 2–3×/month	15 (39.5%)	60 (23.3%)	0.033 *C
Exercised > 60 min/day for weight control	3 (7.9%)	4 (1.6%)	0.017 *C
BMI discrepancy (Actual – Ideal) vs. DE risk			
Actual BMI < Ideal BMI	7 (18.4%)	94 (36.6%)	0.028 *C
Actual BMI > Ideal BMI	31 (81.6%)	163 (63.4%)	

a: independent sample t test; C: chi-square test; * p-value < 0.05 indicates statistical significance

## Discussion

In this cross-sectional study of female students at Birzeit University, 12.9% were identified as being at risk of disordered eating behaviors (DEBs), and BSQ-16B scores showed a moderate positive correlation with EAT-26 scores. The prevalence of DEB risk in our sample was notably lower than reports from similar populations in the region: for example, 28.6% of female students at An-Najah National University were at risk according to the EAT-26, 20% of female university students in the United Arab Emirates were identified as at risk, and 17% of Lebanese medical students were classified as high risk [13, 20, 21]. The lower prevalence observed at Birzeit may reflect differences in sample size, recruitment methods, awareness and stigma around eating problems, socio-cultural norms, and broader environmental factors that influence eating behaviors. Together, these findings suggest that body image dissatisfaction is an important correlate of DEB risk in this understudied Palestinian university population.

Our primary finding was a moderate, positive correlation between the EAT-26 and BSQ-16 scores (*r* = 0.535, *p* = 0.001). This pattern aligns with prior research: for example, Balhara et al. [22] found a strong positive correlation (*r* ≈ 0.58, *p* < 0.001) between EAT-26 and BSQ scores in Indian nursing students [22], and another cross-sectional study conducted among Spanish female university students showed that body satisfaction assessed by BSQ was negatively associated with disordered eating behaviors. This means that as body satisfaction decreases, disordered eating practices increase (*r*=− 0.64, *p* < 0.001) [23].

We examined various socio-demographic and behavioral factors in relation to disordered eating risk (Table 3). Notably, higher actual BMI was significantly associated with eating disorder risk (*p* < 0.001). This mirrors findings from Gulf-region research: for example, Musaiger et al. reported that 81% of obese Kuwaiti female students were dissatisfied with their weight versus only 30% of non-obese students [24], and those obese students were more likely to engage in unhealthy weight-control behaviors. This suggests overlapping risk factors for obesity and disordered eating, chiefly body dissatisfaction. Previous history of disordered eating was also significantly linked with current EAT-26 risk status (*p* = 0.019). This association may indicate that maladaptive eating attitudes and behaviors can persist or recur over time. However, it is important to note that our study assessed self-reported DEBs and not clinically diagnosed eating disorders.

Other important factors included smoking, social media influence, and weight-loss attempts. Smokers were more common in the at-risk group (23.7% vs. 10.5% non-risk), suggesting a possible link between smoking and unhealthy weight-control methods. This finding is consistent with evidence suggesting that some young women use smoking as a perceived appetite-suppression or weight-control strategy [25]. This aligns with previous research indicating that smoking is sometimes used as a coping strategy for managing stress and suppressing appetite, increasing their basal metabolic rate, particularly among young women with body dissatisfaction or eating disorders [25].

Regarding social media, participants who reported a greater influence of social media on their body image showed higher disordered eating risks (*p* = 0.017) and increased body dissatisfaction (*p* = 0.031). The widespread role of social networking in promoting idealized body types (e.g., ultra-thin figures) can lead to the internalization of unrealistic standards, fueling body image issues and unhealthy weight-control behaviors. In fact, research indicates that heavy social media use, particularly appearance-focused content, is associated with greater body dissatisfaction and eating disorder symptoms among young women [23]. A cross-sectional study conducted on college students from Cordoba University found that the thin-ideal appears to be reinforced through social media, which was linked to higher levels of body dissatisfaction and disordered eating attitudes (p-value < 0.05) [23]. Another cross-sectional study involving Chinese and Japanese female college students showed a significant positive correlation between social media use and eating disorder risk (*r* = 0.292, *p* < 0.01) [26].

The association between weight-loss behaviors and ED risk was striking. Participants who engaged in dieting, excessive exercising, meal skipping, or unregulated “fad” methods in the past year were significantly more likely to be at high risk (*p* = 0.001). In our risk group, 55.3% used vigorous exercise and 73.7% used dieting for weight loss. This finding is consistent with previous reports: Iranian students engaging in dieting/skipping meals showed higher disordered eating scores; similarly, female students in Pakistan, UAE, and Gaza who attempted weight loss (through dieting, medication, or reduced meal frequency) also had elevated disordered eating symptoms. We did not systematically assess specific diets or products, but such patterns underscore the convergence of common weight-control attempts with eating disorder risk [14, 27, 28].

Regarding eating disorder symptoms (EAT-26 behavioral items), certain behaviors were significantly more common in the risk group. Binge eating (≥ 2–3 times/month) and daily excessive exercise (≥ 60 min) to control weight were both significantly associated with risk (*p* = 0.033 and *p* = 0.017, respectively). These behaviors are well-recognized markers of eating pathology. Other behaviors (self-induced vomiting, laxative or diet pill use, or > 9 kg weight loss) did not show statistical differences between groups. However, a notable minority of all participants still reported such behaviors (e.g. 17.6% vomiting, 18.3% laxative use) regardless of EAT-26 score. This suggests that even students who do not screen as “high-risk” on the EAT-26 may engage in unhealthy weight-control behaviors. According to EAT-26 guidelines, endorsement of any such behavioral item flags potential risk and warrants further evaluation Therefore, the presence of these behaviors remains a serious concern, even when not captured by the total score cut-off. Reporting bias may also play a role, given cultural sensitivities around eating disorders.

We also assessed BMI discrepancy (difference between actual BMI and self-perceived ideal BMI) in relation to EAT-26 and BSQ categories. Participants who perceived their ideal BMI as lower than their actual (i.e. they wished to lose weight) were significantly more likely to fall in the high-risk group (*p* = 0.028). Conversely, those whose actual BMI was below their ideal (wishing to gain weight) tended to be in the non-risk group. This reflects a well-known pattern: individuals who consider themselves overweight are more prone to engage in restrictive or compensatory eating behaviors.

We found a similar pattern for BSQ: Participants whose actual BMI exceeded their perceived ideal BMI demonstrated higher BSQ-16B scores, indicating greater body-shape dissatisfaction, while those wanting to gain weight reported no concerns. However, other studies indicate that underweight individuals can also experience body dissatisfaction when they feel too thin. One reason we may not have captured this is that the BSQ-16 focuses on fear of gaining weight or being “too fat.” Students desiring weight gain may feel dissatisfied in a different way (e.g. feeling too small), which BSQ does not measure. This suggests a limitation of our body image tool: it may under-detect dissatisfaction in those seeking a larger body. A more nuanced instrument might be needed to assess body image across the full spectrum of weight ideals.

Our study has several limitations. First, the cross-sectional design precludes any causal inference regarding the relationship between BID and DEBs. Second, all data were self-reported, including weight and height, which may have introduced reporting bias and affected the accuracy of BMI-related analyses. Third, the EAT-26 is a screening instrument for DEB risk and possible eating disorder symptoms, but it does not establish a clinical diagnosis of an eating disorder. Fourth, convenience sampling limits the generalizability of the findings, and because the sample size was calculated primarily to estimate DEB prevalence, the study was not specifically powered for inferential analyses examining associations between BID and DEBs or other correlates. Fifth, the BSQ-16B was translated and culturally adapted for this study but was not formally validated in Palestinian university students, which may have affected the precision of BID assessment. Finally, we did not adjust for potentially important confounders such as depression, anxiety, self-esteem, or detailed dietary intake.

Additionally, although the BSQ-16B was culturally adapted, validated Arabic cutoff values for categorical body-shape concern classifications are limited.

Despite these limitations, this study has several strengths. It addresses an important evidence gap in a Palestinian university population, uses the Arabic EAT-26, a previously validated screening tool, and examines the relationship between DEBs, BID, and BMI discrepancy in the same sample. Future research in Palestine should include larger and more diverse student samples, formal validation of Arabic body image instruments, and longitudinal or mixed-methods designs to better understand temporal relationships and cultural influences on DEBs and BID.

## Conclusion

In conclusion, 12.9% of female students at Birzeit University were identified as being at risk of DEBs, and BID showed a moderate positive association with DEB risk. Although the observed prevalence was lower than that reported in some regional studies, unhealthy weight-control behaviors were also reported outside the DEB-risk group. These findings support the need for screening for DEBs and body image concerns among female university students in Palestine.

## Data Availability

Data are available from the corresponding author upon reasonable request.
